# Blood-based DNA methylation as biomarker for breast cancer: a systematic review

**DOI:** 10.1186/s13148-016-0282-6

**Published:** 2016-11-14

**Authors:** Qiuqiong Tang, Jie Cheng, Xue Cao, Harald Surowy, Barbara Burwinkel

**Affiliations:** 1 Molecular Biology of Breast Cancer, Department of Gynecology and Obstetrics, Ruprecht-Karls-Universitaet Heidelberg, Heidelberg, Germany; 2 Division of Molecular Epidemiology (C080), German Cancer Research Center (DKFZ), Heidelberg, Germany

**Keywords:** DNA methylation, Breast cancer, Blood-based biomarker, Systematic review

## Abstract

Multiple studies have investigated global DNA methylation profiles and gene-specific DNA methylation in blood-based DNA to develop powerful screening markers for cancer. This systematic review summarizes the current evidence on methylation studies that investigated methylation level of blood-derived DNA of breast cancer (BC) patients in comparison to healthy controls by conducting a systematic literature review in PubMed and Web of Science. Essential results, such as methylation levels of BC cases and healthy controls, *p* values, and odds ratios, were extracted from these studies by two investigators independently. Overall, 45 publications met the inclusion criteria for this review. DNA from whole blood, as well as cell-free DNA (cfDNA) from serum or plasma, was used in these studies. The most common method used for measuring global DNA methylation was the investigation of repetitive elements as surrogates and the application of array-based genome-wide methylation analysis. For measuring gene-specific methylation level, methylation-specific PCR and pyrosequencing were the most frequently used methods. Epigenome-wide blood DNA hypomethylation in BC patients were reported in several studies; however, the evidence is still not conclusive. The most frequently investigated gene in whole blood was *BRCA1*, which was found more frequently methylated in patients compared to controls. *RASSF1A* was the most widely investigated gene in cfDNA of serum or plasma, which was also found more frequently methylated in patients compared to controls. Several of the eligible studies reported the associations of global hypomethylation and increased BC risk. Studies investigated associations between gene-specific methylation and BC risk, while got heterogeneous results. But two studies reported that hypermethylation of *ATM* gene was associated with increased BC risk, which suggest the potential use of this gene for BC risk stratification. Overall, our review suggests the possibility of using blood-based DNA methylation marker as promising marker for BC risk stratification, as several studies found associations between certain methylation level in blood and BC risk. However, so far, the evidence is still quite limited. Optimal markers are yet to be developed and promising results needed to be validated in prospective study cohorts and tested in large screening populations.

**Electronic supplementary material:**

The online version of this article (doi:10.1186/s13148-016-0282-6) contains supplementary material, which is available to authorized users.

## Background

Breast cancer (BC) is the most common malignancy among women worldwide [1, 2]. The prognosis of this disease mainly depends on its early detection, which currently to a major part relies on mammography. Early detection of this disease can also be facilitated by new diagnostic biomarkers. The current Food and Drug Administration (FDA)-approved blood-based biomarkers for BC, such as CA15-3 and CA27-29, are solely recommended for the monitoring of disease relapse and treatment efficacy, rather than diagnosis [3, 4]. Specific gene mutation tests, such as *BRCA1/2* mutation analysis, are currently only used for screening of hereditary BC cases, which constitute only about 5–10% of total BC cases [5, 6]. For women at normal risk of developing BC, many national organizations recommend screening mammography for older women. In the USA, screening mammography is recommended every 2 years for women at age between 50 and 74 [7]. However, the present screening method is criticized for both low sensitivity [8] and disadvantages due to over-diagnosis [9, 10]. Thus, alternative approaches for BC detection or risk stratification are clearly needed.

Both global hypomethylation and silencing of tumor suppressor genes through promoter hypermethylation can come along with tumor development, and both have been recognized as common hallmarks of many cancers [11]. Similar alterations can also be measured in blood-derived DNA, which suggests the possibility of blood-based DNA methylation markers to serve as new screening markers or markers for risk stratification [12, 13]. To date, a considerable number of studies on DNA methylation in cancer have used DNA obtained from blood (whole blood or white blood cells) or cell-free DNA (cfDNA) isolated from serum or plasma, with the assessment of differences in methylation levels between BC patients and cancer-free healthy controls, to identify methylation markers [14–22]. A substantial number of studies concluded that BC patients and healthy controls exhibit differential DNA methylation patterns in peripheral blood. However, numerous further studies have reported controversial findings, and clear evidence is still lacking whether DNA methylation changes could serve as biomarker for BC diagnosis or risk stratification.

The aim of this review is to summarize the current evidence on DNA methylation-associated biomarkers for BC risk evaluation or early detection, by performing a comprehensive systematic review of published DNA methylation studies in blood-derived DNA of BC patients in comparison to healthy controls. From each eligible study, we extracted essential information, such as age of study subjects, sample size, applied methylation detection methods, methylation levels of patients and healthy controls, *p* values for methylation differences, and odds ratios (ORs), in order to gain insights into the currently accumulated evidence regarding the use of DNA methylation markers for potential future screening tests.

## Methods

### Search strategy

A systematic literature search was performed to identify studies assessing DNA methylation changes in blood as biomarkers for risk or early detection of BC. PubMed and ISI Web of Knowledge were searched for eligible articles until 31 January 2016. The following combination of keywords was used: [breast] (and) [cancer (or) neoplasm (or) carcinoma (or) adenoma (or) malignancy (or) adenoma] (and) [DNA methylation (or) methylated (or) hypermethylation (or) hypomethylation] (and) [risk (or) detection (or) diagnosis] (and) [serum (or) blood (or) plasma (or) white blood cell]. The literature search was limited to studies focusing on humans and published in English.

### Eligibility criteria

Duplicate articles were removed upon combining the retrieved publications from the two databases. A first round of selection was conducted by reviewing the titles and abstracts. Only full-text reports of original studies were included, thus meeting abstracts, reviews, and editorials were excluded. Articles not focusing on DNA methylation changes in blood in the context of BC detection/diagnosis/risk prediction were excluded, including studies that analyzed (1) DNA methylation markers in tissue samples, (2) DNA methylation as prognostic markers of BC or predictive markers for BC treatment efficacy, and (3) DNA isolated from collected CTC cells.

After the first round examination, we conducted a full-text review for the remaining articles. In addition, studies that did not include healthy female controls, for example, only with benign breast disease patients, were not considered. Studies were also excluded if the information regarding methylation levels of BC cases and healthy controls or ORs were not reported or could not be extracted from published data, for example, studies that solely presented results by heatmaps or reported the methylation levels of a combination of specific loci/genes. Cross-referencing was used as a possible source for identifying studies related to the present topic.

### Data extraction and statistical analysis

Eligible studies were included in the data extraction procedure, which was conducted independently by two investigators (Q. Tang and J. Cheng) with a standardized data extraction form. The following variables were extracted: first author, publication year, study design, age of study subjects, DNA source (whole blood, serum, or plasma), DNA methylation detection method, the type of measured DNA methylation (global or gene/locus specific), and essential results (methylation levels of cases and controls, ORs, *p* values). Any disagreement was resolved by further review and discussion among the coauthors. In case methylation levels were not explicitly reported, the information was extracted from available tables and figures to the possible extent. If not presented in the articles, *p* values for methylation differences between BC cases and healthy controls were calculated by Fisher’s exact test. Reporting of data follows the PRISMA statements [23].

## Results

### Literature overview

**Fig. 1:**
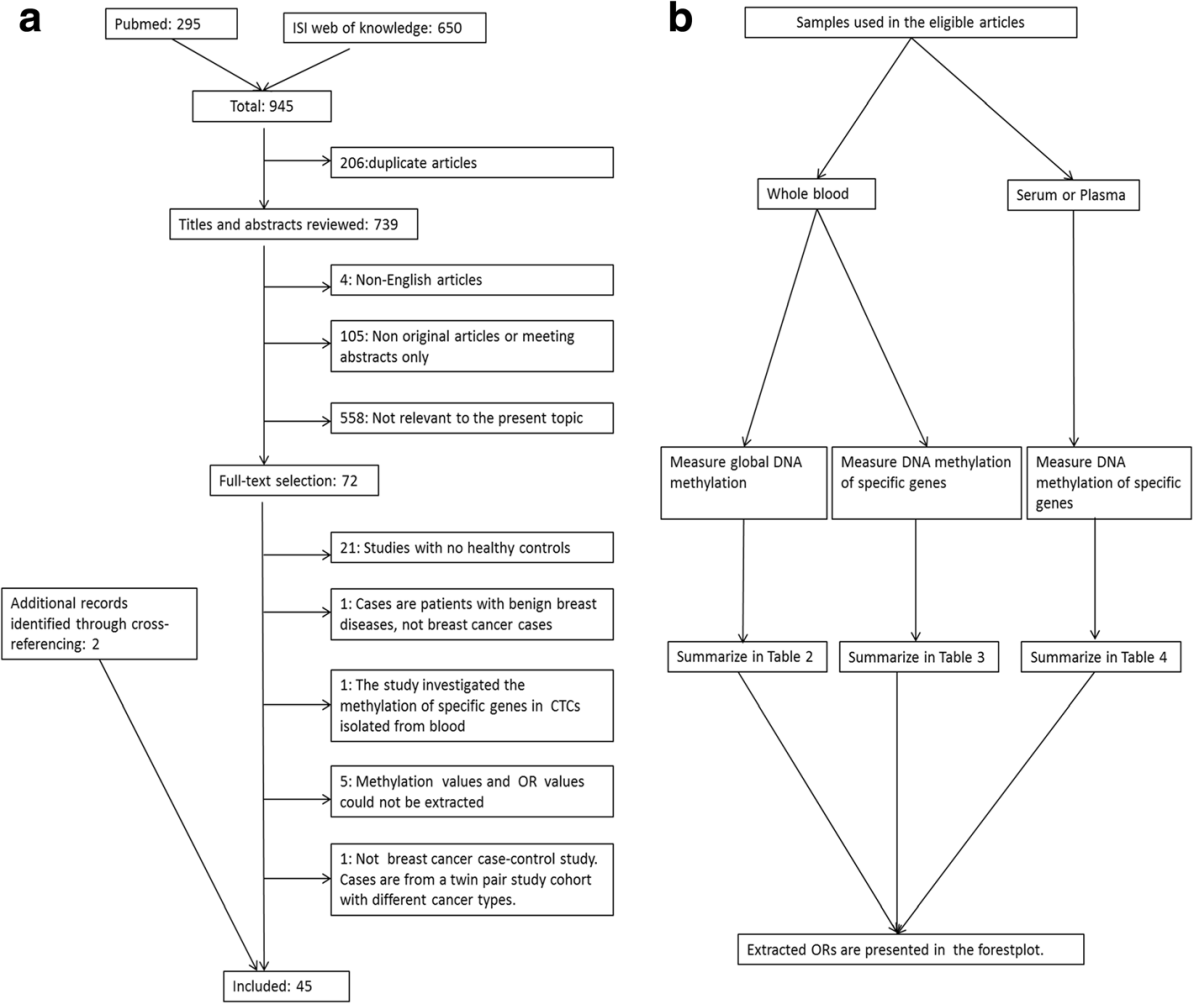
**a** Flow diagram of the literature search process (search until 30.01.2016) and **b** summarize strategy of the review

The process of the systematic literature search is displayed in Fig. 1a. Briefly, the primary search in PubMed and Web of knowledge identified 945 articles, of which 206 were duplicate articles. After excluding non-eligible articles (see Fig. 1a and Additional file 1: Supplementary materials), 45 articles could be included in this review, including 26 articles used DNA isolated from whole blood [14–21, 24–41], two articles used DNA isolated from both whole blood and plasma [42, 43], six articles used DNA isolated from plasma [44–49], and 11 articles used DNA isolated from serum [50–60] (Table 1). For the studies that used serum or plasma as DNA source, four of them used two centrifugation steps to get serum or plasma [42, 45, 47, 48] and the rest used one centrifugation step or sample processing procedures are not available (Table 1). The included articles were published between 2004 and 2015.

**Table 1:** Characteristics summary of the 45 eligible studies

Number	First author	Year	Country	DNA source	Sample treatment	Measurement	Methylation levels available	Odds ratio estimation available
1	Widschwendter M	2008	Germany	Blood	–	Gene-specific methylation	Yes	Yes
2	Snell C	2008	Australia	Blood	–	Gene-specific methylation	Yes	No
3	Ito Y	2008	UK	Blood	–	Gene-specific methylation	Yes	Yes
4	Flanagan. JM	2009	UK	Blood	–	Gene-specific methylation	Yes	Yes
5	Choi JY	2009	USA	Blood	–	Global DNA methylation	Yes	Yes
6	Cho YH	2010	Turkey	Blood	–	Both global DNA methylation and gene-specific methylation	Yes	No
7	Hoffman AE	2010	Connecticut	Blood	–	Gene-specific methylation	No	Yes
8	Wong EM	2011	Australia	Blood	–	Gene-specific methylation	Yes	Yes
9	Iwamoto T	2011	Japan	Blood	–	Gene-specific methylation	Yes	Yes
10	Brennan K	2012	Australia, New Zealand, UK, and Europe	Blood	–	Both global DNA methylation and gene-specific methylation	Yes	Yes
11	Xu X	2012	USA	Blood	–	Global DNA methylation	Yes	Yes
12	Bosviel R	2012	France	Blood	–	Gene-specific methylation	Yes	No
13	Wu HC	2012	USA	Blood	–	Global DNA methylation	Yes	Yes
14	Delgado-Cruzata L	2012	USA	Blood	–	Global DNA methylation	Yes	Yes
15	Kitkumthorn N	2012	Thailand	Blood	–	Global DNA methylation	Yes	No
16	Hajikhan Mirzaei M	2012	Iran	Blood	–	Gene-specific methylation	Yes	No
17	Askari M	2013	India	Blood	–	Gene-specific methylation	Yes	Yes
18	Severi G	2014	Australia	Blood	–	Global DNA methylation	Yes	Yes
19	Yang RX	2014	Germany	Blood	–	Gene-specific methylation	Yes	Yes
20	Kuchiba A	2014	Japan	Blood	–	Global DNA methylation	Yes	Yes
21	Gupta S	2014	Poland	Blood	–	Gene-specific methylation	Yes	Yes
22	DeRoo LA	2014	USA	Blood	–	Global DNA methylation	No	Yes
23	van Veldhoven K	2015	Italy	Blood	–	Global DNA methylation	Yes	Yes
24	Cho YH	2015	USA	Blood	–	Gene-specific methylation	Yes	Yes
25	Yari K	2015	Iran	Blood	–	Gene-specific methylation	Yes	No
26	Harrison K	2015	Europe	Blood	–	Gene-specific methylation	Yes	Yes
27	Zmetakova I	2013	Slovakia	Blood and plasma	1000*g* for 10 min + 1000*g* for 10 min	Gene-specific methylation	Yes	No
28	Enders KN	2014	China	Blood and plasma	na	Gene-specific methylation	Yes	No
29	Hoque MO	2006	Senegal	Plasma	2200 rpm for 10–15 min	Gene-specific methylation	Yes	No
30	Papadopoulou E	2006	Greece	Plasma	2000 rpm for 10 min + 2000 rpm for 10 min	Gene-specific methylation	Yes	No
31	Yazici H	2009	USA	Plasma	na	Gene-specific methylation	Yes	No
32	Radpour R	2011	Switzerland	Plasma	16,006*g* for 10 min + full speed 10 min	Gene-specific methylation	Yes	No
33	Enders KO Ng	2011	China	Plasma	1600*g* for 10 min + 16,000*g* for 10 min	Gene-specific methylation	Yes	No
34	Chimonidou M	2013	Greece	Plasma	2000*g* for 10 min	Gene-specific methylation	Yes	No
35	Dulaimi E	2004	Pennsylvania	Serum	na	Gene-specific methylation	Yes	No
36	Martinez-Galan J	2008	Spain	Serum	2000*g* for 10 min	Gene-specific methylation	Yes	No
37	Van der Auwera I	2009	Belgium	Serum	2000*g* for 10 min	Gene-specific methylation	Yes	No
38	Chen Z	2009	China	Serum	1000*g* for 10 min	Gene-specific methylation	Yes	No
39	Zurita M	2010	Spain	Serum	2000*g* for 10 min	Gene-specific methylation	Yes	No
40	Ahmed IA	2010	Germany	Serum	2000*g* for 10 min	Gene-specific methylation	Yes	No
41	Brooks JD	2010	USA	Serum	na	Gene-specific methylation	Yes	No
42	Kim JH	2010	Korea	Serum	na	Gene-specific methylation	Yes	No
43	Kloten V	2013	Germany	Serum	2000*g* for 10 min	Gene-specific methylation	Yes	No
44	Swellam M	2015	Egypt	Serum	1600*g* for 15 min	Gene-specific methylation	Yes	No
45	Liu LM	2015	China	Serum	na	Gene-specific methylation	Yes	No

*na* not available

Among all eligible studies, only 11 studies investigated global DNA methylation. This was always done in DNA isolated from whole blood (Table 1). The majority of studies measured gene- or locus-specific DNA methylation levels, in DNA isolated either from whole blood or in cfDNA isolated from serum or plasma (Table 1). To get a better overview of global DNA methylation changes, as well as the differentially methylated genes between BC patients and healthy controls, we summarize these studies separately, as shown in Fig. 1b.

### Global DNA methylation in peripheral blood of BC cases and controls

As shown in Table 2, a total of 15 studies from 11 literatures evaluated global DNA methylation levels in whole blood by different strategies. These included using mean methylation intensities of all Infinium HumanMethylation450K (450 K) probes (*β* value) as global DNA methylation levels, measuring the percentage of methylated DNA by luminometric methylation assay (LUMA) and the concentration of 5-methyldeoxycytosine (5-mdC) by liquid chromatography-mass spectrometry (LC-MS) or measuring the methylation of repetitive DNA elements (i.e., LINE-1, Alu, or Sat2) by pyrosequencing or the MethyLight assay as surrogates of global DNA methylation levels. Among them, four nested case–control studies [17–19, 38] used prospectively collected samples of BC cases and healthy controls, while the remaining studies used samples collected at diagnosis or shortly after diagnosis and healthy controls [17, 26, 27, 29, 31–33, 36]. Case number of these studies was between relative large (over 100 subjects for each group), except the studies by Kitkumthorn N et al. (with 36 cases) [33] and Cho YH et al. (with 40 cases and 40 controls) [27]. Cases and controls used in these 14 studies were number- and age-matched.

**Table 2:** Global DNA methylation in peripheral blood of breast cancer cases and healthy controls

Measurements	Author, year [ref]	Study design	Assay (value)	Case no./control no.	Case age/control age (y)^a^	Meth (case)	Meth (control)	*p* value	Main findings
*β* value	van Veldhoven K, 2015 [18]	Nested case–control	450 K (EPIC cohort) (mean + SD)	162/162	54.4/54.2	53.00 ± 0.39	53.18 ± 0.35	1.82E−05	Epigenome-wide hypomethylation of DNA in samples from EPIC cohort.
			450 K (NOWAC cohort) (mean + SD)	168/168	55.4/55.4	54.02 ± 0.45	54.02 ± 0.41	0.79	
			WBGS (BGS cohort) (mean)	548/548	52/52	48.12	48.3	na	
	Severi G, 2014 [19]	Nested case–control	450 K (mean + SD)	420/420	64/64	51.86 ± 1.00	51.95 ± 1.01	0.006	Epigenome-wide hypomethylation of DNA in BC patients.
LUMA	Kuchiba A, 2014 [36]	Case–control	LUMA (% DNA meth)	384/384	54.1/53.9	68.9 ± 3.5	70.2 ± 3.4	<0.01	Global genomic hypomethylation in BC patients.
	Xu X, 2012 [29]	Case–control	LUMA (%)	1055/1101	na/na	57.3 ± 15.7	52.4 ± 16.7	<0.0001	Global promoter hypermethylation in patients.
	Delgado-Cruzata L, 2012 [32]	Case–control	LUMA (%)	263/321	49.5/48.0	67.1 ± 7.6	67.5 ± 7.3	>0.05	LUMA DNA methylation levels were similar between cases and controls.
5-mdC	Choi JY, 2009 [26]	Case–control	LC-MS (test set) (mean)	19/18	35–75/35–75	3.98	4.33	0.001	Hypomethylation of 5-mdC in BC patients.
			LC-MS (validation set) (mean)	176/173	35–75/35–75	4.18 ± 0.34	4.38 ± 0.36	<0.001	
LINE-1	Kitkumthorn N, 2012 [33]	Case–control	COBRA (%)	36/144	50.28/48.67	*40*	*42*	>0.05	No significant differences in LINE-1 methylation between BC cases and healthy controls.
	Xu X, 2012 [29]	Case–control	Pyrosequencing (mean)	1064/1100	na/na	78.8	78.8	0.94	As above.
	Brennan K, 2012 [17]		Pyrosequencing (mean and IQR)						As above.
		Case–control	BGS cohort	241/242	54/54	79.0 (78.1–79.9)	79.0 (77.9–80.1)	0.96	
		Case–control	EPIC cohort	232/263	52/52	75.2 (73.9–76.3)	75.1 (73.9–76.3)	0.89	
		Nested case–control	KConFab cohort	153/218	50/60	76.6 (75.2–77.6)	76.0 (74.3–78.0)	0.2	
	Wu HC, 2012 [31]	Case–control	MethyLight (%)	265/333	49.5/48.0	107.4 ± 63.6	108.5 ± 59.1	>0.05	As above.
			Pyrosequencing (mean)	279/340	49.5/48.0	74.5 ± 3.0	74.5 ± 2.6	>0.05	
	Cho YH, 2010 [27]	Case–control	MethyLight (%)	40/40	50.8/48.3	*70*	*78*	>0.05	As above.
	Choi JY, 2009 [26]	Case–control	Pyrosequencing (mean)	19/18	35–75/35–75	74.7	73.9	0.176	As above.
	Deroo LA, 2014 [38]^b^	Nested case–control	Pyrosequencing	294/646	57.9/na	na	na	na	As above.
Sat2	Wu HC, 2012 [31]	Case–control	MethyLight (%)	266/333	49.5/48.0	41.3 ± 24.4	43.5 ± 32.9	>0.05	No significant differences in Sat2 methylation between BC cases and healthy controls.
	Cho YH, 2010 [27]	Case–control	MethyLight (%)	40/40	50.8/48.3	*125*	*150*	0.01	Hypomethylation of Sat2 inpatients.
Alu	Wu HC, 2012 [31]	Case–control	MethyLight (%)	266/334	49.5/48.0	95.5 ± 36.6	98.7 ± 51.5	>0.05	No significant differences in Alu methylation between BC cases and healthy controls.
	Cho YH, 2010 [27]	Case–control	MethyLight (%)	40/40	50.8/48.3	*58*	*61*	>0.05	As above.
[3H]-methyl	Delgado-Cruzata L, 2012 [32]	Case–control	[3H]-Methyl acceptance assay	233/295	49.6/48.2	97,111 ± 76,348	88,030 ± 70,841	<0.05	Global genomic hypomethylation in BC patients (more [3H]-methyl acceptance).

The numbers in italic are extracted from boxplot or scatter plots

*450K* Infinium HumanMethylation 450K Beadchips, *WGBS* whole genome bisulfite sequencing, *LUMA* luminometric methylation assay, *COBRA* combined bisulfite restriction analysis, *5-mdC* 5-methyldeoxycytosine, *na* not available

^a^Age indicates mean age or range

^b^The mean DNA methylation level of BC cases and controls is not available; the study only reported the results of the quartile analysis

As shown in Table 2, studies of van Veldhoven and Severi reported epigenome-wide hypomethylation of blood DNA in BC patients compared to controls, even that van Veldhoven et al. [18] observed lower methylation in BC cases in one of their study cohorts, but not in another two study cohorts. Three studies [29, 32, 36] measured the global methylation content by LUMA assay but obtained heterogeneous results. Specifically, Kuchiba et al. [36] observed an increased global blood DNA methylation in BC patients, while Xu et al. [29] reported a decrease and Delgado-Cruzata et al. [32] found no significant methylation differences between BC cases and controls. Choi JY et al. [26] observed significant lower level of 5-mdC in patients compared to controls. Interestingly, nine studies from seven articles [17, 26, 27, 29, 31, 33, 38] evaluated the methylation level of LINE-1 repeats with different detection methods, but almost all of them reported that there were no significant difference in LINE-1 methylation between BC cases and controls (Table 2). Studies investigating Sat2 and Alu repetitive elements also revealed inconsistent results (Table 2). Delgado-Cruzata L et al. [32] observed significant higher [3H]-methyl acceptance (lower DNA methylation) in patients than in controls.

Overall, the evidence of global DNA hypo- or hypermethylation in blood DNA of BC cases is so far limited and not conclusive. As shown in Table 2, less than half of these studies reported significant global hypomethylation in blood DNA of BC patients (Table 2) and the overall methylation difference between BC cases and controls are relative small (effect size varied from 0.013 to 0.25). This could be due to the complicated epigenetic background of DNA isolated from whole blood as well as the still high variability of quantitative DNA methylation detection methods. In addition, the eligibility of LINE-1 as surrogate for global DNA methylation level might be limited, as nine studies observed no significant difference of LINE-1 methylation between cases and controls.

**Fig. 2:**
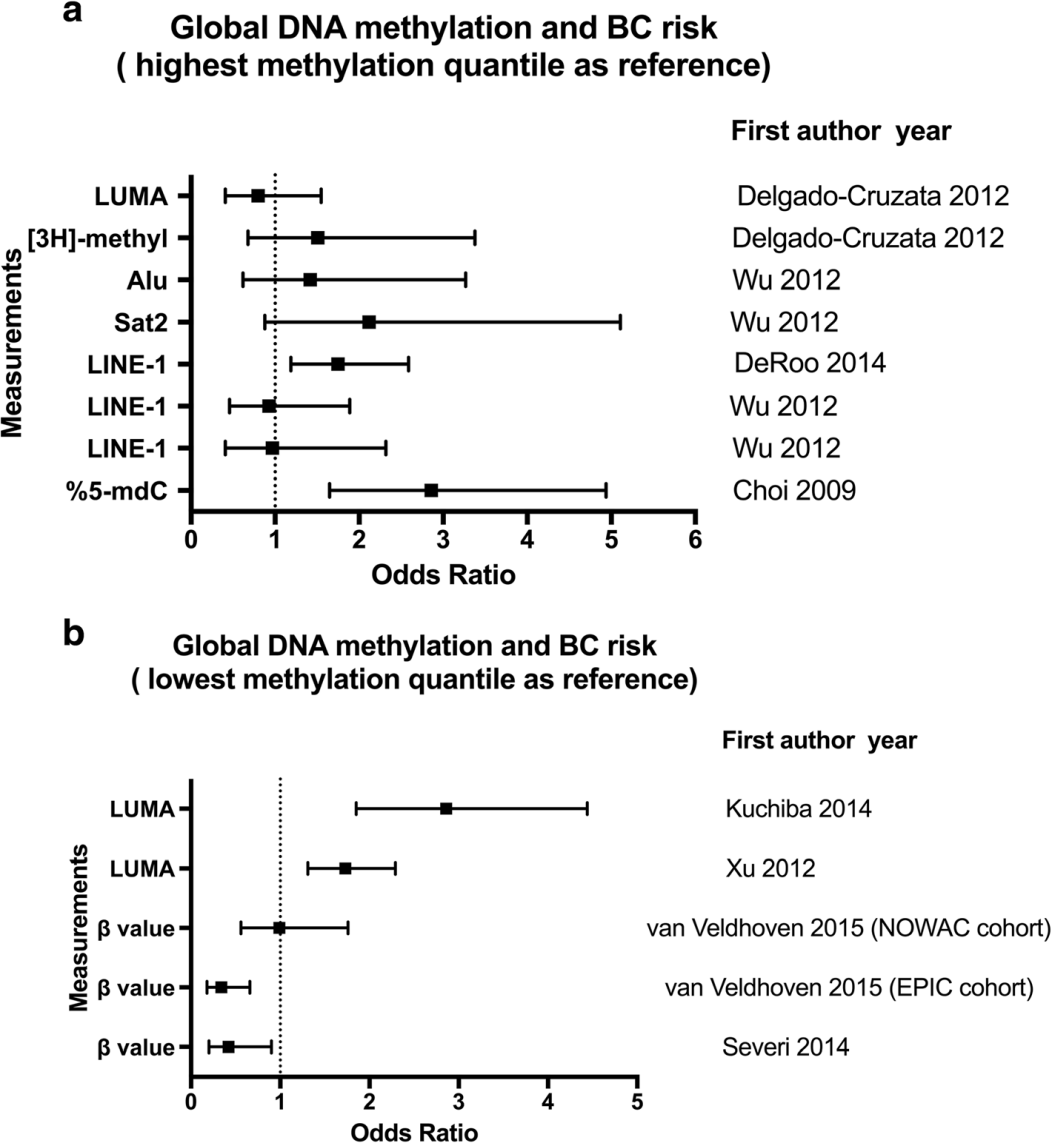
Associations of global DNA methylation in blood and BC risk. **a** Studies used the highest methylation quantile as reference. **b** Studies used the lowest methylation quantile as reference

Some studies also investigated the associations between blood DNA methylation levels and BC risk by quantile analysis, comparing the risk of women in the highest quantile and that of women in the lowest quantile (Fig. 2a) or vice versa (Fig. 2b). As shown in Fig. 2a, Delgado-Cruzata and coauthors concluded that there was no significant association between global DNA methylation levels detected by LUMA assay and [3H]-methyl acceptance assay and BC risk [32]. Wu HC et al. [31] and DeRoo LA et al. [38] evaluated possible associations between the methylation level of repetitive elements (LINE-1, Alu, or Sat2) of blood DNA and BC risk, but also with inconsistent results. Choi et al. used the amounts of 5-mdC as surrogates for global DNA methylation in blood and reported that women representing the lowest 5-mdC quantile had a higher risk of BC (2.81, 95% CI 1.65–4.94), compared with women of the highest quantile [26]. As shown in Fig. 2b, Xu et al. [29] and Kuchiba et al. [36] revealed a positive association between LUMA methylation level and BC risk. Three large prospective studies were reported in two articles. Here, the global DNA methylation was investigated by 450K methylation arrays. Mean *β* values across the whole genome were calculated and used as global DNA methylation level [18, 19]. For women in the highest quantile compared to women in the lowest methylation quantile, the ORs (95% CI) were 0.34 (0.18–0.66) (EPIC cohort) and 0.99 (0.56–1.76) (NOWAC cohort) in the study of van Veldhoven et al. [18], and the ORs were 0.42 (0.20–0.90) in the study of Severi and coauthors [19]. This suggests that hypomethylation in whole blood might be associated with an increased risk of BC, even the abovementioned results are inconclusive.

Overall, the association between global DNA methylation and BC risk is still unclear, as both positive association and negative association were reported.

### Gene-specific methylation in whole blood DNA of BC cases and controls

Table 3 and Additional file 1: Table S1 list all the studies that examined the methylation levels of specific gene loci in whole blood DNA of BC cases and healthy controls. All of these studies were case–control studies. The number of cases varied from only seven to 1021. The most frequently used methods for detection of gene-specific methylation levels were MethyLight and pyrosequencing. *BRCA1* was investigated in seven studies and thus the most frequently investigated gene [16, 20, 24, 27, 30, 37, 39]. Importantly, all these studies reported a rather higher frequency of methylated *BRCA1* in BC cases than in healthy controls, although the differences were only statistically significant in four studies [16, 20, 24, 37]. *ATM* was investigated in two studies [15, 17], and both of them observed hypermethylation of *ATM* in BC patients. Methylation levels of *IGF2* [25, 41], *CDH1* [39, 42], *SYK* [14, 42], *RARB* [27, 39], *APC* [27, 42], and *RASSF1A* [27, 42] were found similar between BC patients and controls in two or more studies. Methylation of *ESR* [14, 42] and *TIMP3* [14, 42] were also determined in more than one study, while the methylation differences of these genes between blood DNA of BC cases and controls were not conclusive. Other genes investigated in only one study were summarized in Additional file 1: Table S1.

**Table 3:** Gene-specific methylation in peripheral blood DNA in breast cancer cases and controls investigated in more than one study

Gene	Author, year [ref]^a^	Assay (value)	Case no./control no.	Case age/control age (y)^b^	Meth (case)	Meth (control)	*p* value	Main findings
*BRCA1*	Cho YH, 2015 [39]	MethyLight (%)	1021/1036	na/na	12	10	>0.05	Higher frequency of methylated *BRCA1* in BC patients was observed in all six studies.
	Gupta S, 2014 [37]	MS-HRM (%)	66/36	48.8/56.1	22.7	5.6	0.03	
	Bosviel R, 2012 [30]	QMSP (%)	902/990	47.1/45.9	47.1 (46.1–48.1)	45.9 (45.0–46.8)	0.08	
	Wong EM, 2011 [20]	MS-HRM (%)	255/169	<40/<40	10.9	3.6	0.004	
	Iwamoto T, 2011 [16]	MSP (%)	200/200	50/50	21.5	13.5	0.045	
	Cho YH, 2010 [27]	MethyLight (%)	40/40	50.8/48.3	8	5	>0.05	
	Snell C, 2008 [24]	MethyLight (%)	7/7	35–51/35–51	42.9	14.3	<0.05	
*ATM*	Brennan K, 2012 [17]	Pyrosequencing ATM (mvp2a)						Hypermethylation of *ATM* (intragenic repetitive element) in BC patients was observed in two studies.
		BGS cohort (mean and IQR)	249/248	54/54	76.8 (70.9–82.7)	76.4 (70.2–80.2)	0.02	
		EPIC cohort (mean and IQR)	235/283	52/52	75.7 (70.0–80.8)	76.1 (70.5–80.6)	0.4	
		KConFab cohort (mean and IQR)	156/210	50/60	81.8 (75.8–86.5)	76.9 (71.6–81.5)	4.87 × 10^−6^	
		Pyrosequencing ATM (mvp2b)						
		BGS cohort (mean and IQR)	248/234	54/54	91.4 (85.6–95.0)	91.0 (87.0–94.8)	0.61	
		EPIC cohort (mean and IQR)	240/287	52/52	92.3 (88.3–95.7)	92.2 (87.3–95.2)	0.36	
		KConFab cohort (mean and IQR)	162/208	50/60	92.3 (82.4–96.5)	92.6 (87.2–96.3)	0.24	
	Flanagan JM, 2009 [15]	Pyrosequencing (mean and IQR)	190/190	62.8/62.8	91.4 (72.8–98.4)	89.8 (53.0–98.0)	0.002	
*IGF2*	Harrison K, 2015 [41]	Pyrosequencing (mean ± SD)	189/363	56/56	48.94 ± 5.61	48.15 ± 5.77	0.123	Two studies reported no significant differences in methylation of *IGF2* between BC cases and healthy controls.
	Ito Y, 2008 [25]	Pyrosequencing (% of loss of methylation)						
		EPIC-Norfolk cohort	228/460	60.5/60.3	6.6	6.3	0.91	
		ABC cohort	338/84	52.6/43.2	5.6	7.1	0.65	
*CDH1*	Cho YH, 2015 [39]	MethyLight (%)	1021/1036	na/na	58	66	>0.05	Three studies observed no significant differences in methylation of *CDH1* between BC cases and controls.
	Zmetakova I, 2013 [42]	Pyrosequencing (mean ± SD)	34/50	41–90/20–78	9.64 ± 2.10	9.02 ± 1.60	0.698	
	Cho YH, 2010 [27]	MethyLight (%)	40/40	50.8/48.3	8	8	>0.05	
*ESR1*	Zmetakova I, 2013 [42]	Pyrosequencing (mean ± SD)	34/50	41–90/20–78	4.09 ± 1.44	3.22 ± 0.86	0.026	Zmetakova I et al. reported higher methylation of *ESR1* in patients, while Widschwendter. M et al. observed no significant difference.
	Widschwendter M, 2008 [14]	MethyLight (%)	320/676	50–74/50–74	12.2	13.5	0.645	
*SYK*	Zmetakova I, 2013 [42]	Pyrosequencing (mean ± SD)	34/50	41–90/20–78	1.15 ± 0.44	1.06 ± 0.24	0.638	Both studies observed no significant differences in methylation of *SYK* between BC cases and controls.
	Widschwendter M, 2008 [14]	MethyLight (%)	320/676	50–74/50–74	2.2	2.4	0.889	
*TIMP3*	Zmetakova I, 2013 [42]	Pyrosequencing	34/50	41–90/20–78	3.65 ± 2.55	2.50 ± 0.81	0.036	Zmetakova I et al. reported higher methylation of *TIMP3* in patients, while Widschwendter. M et al. observed no significant difference.
	Widschwendter M, 2008 [14]	MethyLight (%)	320/676	50–74/50–74	12.5	14.2	0.511	
*RARB*	Cho YH, 2015 [39]	MethyLight (%)	1021/1036	na/na	33	39	>0.05	Two studies reported no significant differences in methylation of *RARB* between BC cases and healthy controls.
	Cho YH, 2010 [27]	MethyLight (%)	40/40	50.8/48.3	10	10	>0.05	
*APC*	Zmetakova I, 2013 [42]	Pyrosequencing (mean ± SD)	34/50	41–90/20–78	1.68 ± 1.04	1.28 ± 0.57	0.082	Two studies reported no significant differences in methylation of *APC* between BC cases and healthy controls.
	Cho YH, 2010 [27]	MethyLight (%)	40/40	50.8/48.3	0	0	>0.05	
*RASSF1A*	Zmetakova I, 2013 [42]	Pyrosequencing (mean ± SD)	34/50	41–90/20–78	1.00 ± 0.00	1.04 ± 0.28	0.475	Two studies reported no significant differences in methylation of *RASSF1A* between BC cases and healthy controls.
	Cho YH, 2010 [27]	MethyLight (%)	40/40	50.8/48.3	8	3	>0.05	

*na* not available

^a^All studies were case–control study

^b^Age indicates mean age or range

**Fig. 3:**
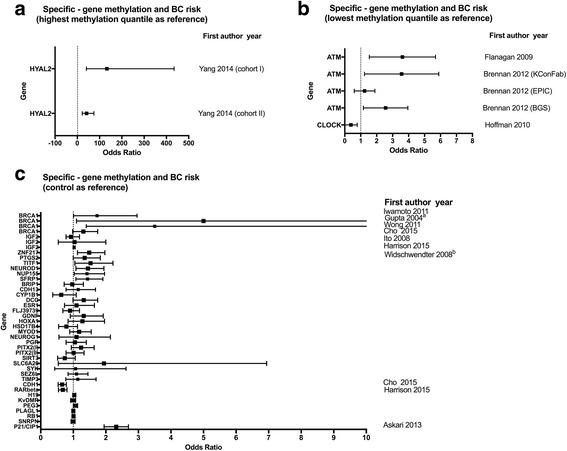
Associations of gene-specific methylation in blood and BC risk. **a** Studies used the highest methylation quantile as reference. **b** Studies used the lowest methylation quantile as reference. **c** Studies used methylation of controls as reference. ^a^The upper limit of 95% CI of the study of Gupta was over ten. ^b^Widschwendter M and coauthors investigated the genes from *ZNF217* to *TIMP3*

Figure 3 shows the associations of gene-specific methylation in blood and BC risk. Yang et al. [21] showed that reduced methylation levels of the *HYAL2* gene were significantly associated with increased BC risk. Specifically, women in the highest quartile of *HYAL2* methylation were reported to have a 41.47-fold (cohort I) and a 132.98-fold (cohort II) increased BC risk, compared with women in the lowest quartile (Fig. 3a). Hypermethylation of *ATM* and increased BC risk were observed in two studies [15, 17]. Here, the lowest methylation quantile was used as reference (Fig. 3b). Hoffman et al. observed a negative association between *CLOCK* methylation and BC risk [28] (Fig. 3b). Widschwendter et al. investigated methylation of a few genes in a case–control study (*n* = 1083) and found that decreased DNA methylation in *NUP155 (I)*, *ZNF217 (II)*, *PTGS2*, *TITF1*, *NEUROD1*, and *SFRP1* are associated with increased BC risk [14] (Fig. 3c). Hypermethylation of *BRCA1* promoter was associated with increased BC risk, which was confirmed in two independent studies [16, 37] (Fig. 3c).

### Gene-specific methylation in cfDNA from serum or plasma of BC cases and controls

Table 4 summarizes all studies that investigated methylation differences of specific genes in serum or plasma DNA of BC cases and healthy controls. Studies conducted by Yazici et al. [46] and Brooks et al. [56] were nested case–control studies and the remaining studies were all case–control studies. Generally, the sample sizes were rather low. Case number varied from 4 to 250. All eligible studies using serum or plasma DNA investigated DNA methylation levels at specific loci, rather than global DNA methylation levels. Further, so far no epigenome-wide study has been performed on cfDNA. This can be explained by the technical difficulties due to the specific characteristics, such as strongly fragmented DNA and reduced DNA integrity especially in cancer cases [61]), and limited amounts of cfDNA that can be isolated from serum or plasma [62–64], and also to uncertainties regarding its origins [65]. The most common method used to measure the methylation levels of specific genes in serum or plasma cfDNA was methylation-specific PCR (MSP) (Table 4 and Additional file 1: Table S2).

**Table 4:** Gene-specific methylation in serum or plasma DNA in breast cancer cases and controls investigated in more than one study

Gene	Author, year [ref]	Sample	Assay (value)	Case no./control no.	Case age/control age (y)^b^	Meth (case)	Meth (control)	*p* value	Main findings
*RASSF1A*	Kloten V, 2013 [58]	Serum	MS-PCR (%)	136/135	33–86/33–86	47.1	25.9	0.004	Higher frequency of methylated *RASSF1A* was observed in eight studies. Studies by Zmetakova I et al. and Brooks JD et al. reported no significant differences in methylation of *RASSF1A* between cases and controls
	Zmetakova I, 2013 [42]	Plasma	Pyrosequencing (mean ± SD)	34/50	41–90/20–78	2.85 ± 3.13	4.02 ± 6.62	0.404	
	Ahmed IA, 2010 [55]	Serum	MSP (%)	26/12	35–73/35–73	69	<10	–	
	Brooks JD, 2010 [56]^d^	Serum	QMSP (%)	50/99	52/51.8	22	17.2	>0.05	
	Kim JH, 2010 [57]	Serum	QMSP (%)	119/125	51/51	32.8	4.8	0.004	
	Yazici H, 2009 [46]^d^	Plasma	MSP (%)	61/39	na/na	18	5	–	
	Hoque M, 2006 [44]	Plasma	QMSP (%)	47/38	44.9/37.3	32	5	0.002	
	Van der Auwera I, 2009 [52]	Serum	QMSP (%)	79/19	62/39	35	0	0.002	
	Papadopoulou E, 2006 [45]	Plasma	Methylight (%)	50/14	na/na	26	0	<0.05	
	Dulaimi E, 2004 [50]	Serum	MSP (%)	34/20	57.4/57.4	56	0	<0.05^c^	
*APC*	Swellam M, 2015 [59]	Serum	MS-PCR (%)	121/66	43/40	93.4	0	<0.0001	Five out of these seven studies reported higher frequency of methylated *APC* in BC patients. Studies by Zmetakova I et al. and Brooks JD et al. reported no significant differences in methylation of APC between cases and controls.
	Zmetakova I, 2013 [42]	Plasma	Pyrosequencing (mean ± SD)	34/50	41–90/20–78	4.41 ± 7.81	2.53 ± 1.56	0.06	
	Radpour R, 2011 [47]	Plasma	EpiTyper assay (mean)	36/30	67/na	0.39^b^	0.19^b^	<0.0001	
	Brooks JD, 2010 [56]^d^	Serum	QMSP (%)	49/96	52/51.8	2	4.2	>0.05	
	Hoque M, 2006 [44]	Plasma	QMSP (%)	47/38	44.9/37.3	17	0	0.008	
	Van der Auwera I, 2009 [52]	Serum	QMSP (%)	79/19	62/39	29	5	0.03	
	Dulaimi E, 2004 [50]	Serum	MSP (%)	34/20	57.4/57.4	29	0	<0.05^c^	
*ESR1*	Zmetakova I, 2013 [42]	Plasma	Pyrosequencing (mean ± SD)	34/50	41–90/20–78	4.18 ± 4.07	5.24 ± 4.33	0.338	Only one study (Matinez-Galan, J) reported higher methylation levels of *ESR1* in BC patients. Others observed no significant methylation differences.
	Zurita M, 2010 [54]	Serum	QMSP (%)	77/34	na/na	0.005^b^	0.085^b^	>0.05	
	Van der Auwera I, 2009 [52]	Serum	QMSP (%)	79/19	62/39	20	10.5	0.33	
	Martinez-Galan J, 2008 [51]	Serum	MSP (%)	106/74	58/42	0.11^b^	0.02^b^	0.011	
*RARB*	Swellam M, 2015 [59]	Serum	MS-PCR (%)	121/66	43/40	95.9	0	<0.0001	Higher frequency of methylated *RARB* was observed except the study conducted by Brooks JD et al.
	Brooks JD, 2010 [56]^d^	Serum	QMSP (%)	45/88	52/51.8	6.7	1.1	>0.05	
	Kim JH, 2010 [57]	Serum	QMSP (%)	119/125	51/51	86.6	6.4	<0.001	
	Hoque M, 2006 [44]	Plasma	QMSP (%)	47/38	44.9/37.3	26	8	0.03	
*GSTP1*	Radpour R, 2011 [47]	Plasma	EpiTyper assay (mean)	36/30	67/na	0.52^b^	0.39^b^	0.003	Two studies reported higher methylation level (Radpour R et al., 2011) or frequency (Hoque M et al., 2006) of *GSTP1* in BC patients. Study by Brooks.J.D observed no significant differences.
	Brooks JD, 2010 [56]^d^	Serum	QMSP (%)	50/99	52/51.8	4	7.1	>0.05	
	Hoque M, 2006 [44]	Plasma	QMSP (%)	47/38	44.9/37.3	26	0	0.0008	
*SFN*	Zurita M, 2010 [54]	Serum	QMSP (%)	77/34	na/na	0.002^b^	0.1^b^	<0.001	Both studies reported higher methlyation of *SFN* in BC patients.
	Martinez-Galan J, 2008 [51]	Serum	MSP (%)	106/74	58/42	0.20^b^	0.075^b^	0.0047	
*BRCA1*	Liu LM, 2015 [60]	Serum	Bisulfite sequencing PCR and MS-HRM (%)	36^a^/30^a^	na/na	10	1.7	<0.05	Both studies reported higher methlyation of *BRCA1* in BC patients
	Radpour R, 2011 [47]	Plasma	EpiTyper assay	36/30	67/na	0.58^b^	0.30^b^	<0.0001	
*CST6*	Chimonidou M, 2013 [49]	Plasma	MSP (%)	73/37	na/na	16.4	0		ChimonidouM et al. reported that *CST6* promoter is highly methylated in cfDNA of breast cancer patients, but not in healthy individuals. Radpour R et al. observed higher methlytion level of *CST6* in BC patients.
	Radpour R, 2011 [47]	Plasma	EpiTyper assay (mean)	36/30	67/na	0.62^b^	0.42^b^	<0.002	
*DAPK*	Ahmed IA, 2010 [55]	Serum	MSP (%)	26/12	35–73/35–73	88	<10%	<0.05	Higher frequency of methylated *DAPK* in patients was observed in both studies.
	Dulaimi E, 2004 [50]	Serum	MSP	34/20	57.4/57.4	35	0	<0.05^c^	
*TIMP3*	Zmetakova I, 2013 [42]	Plasma	Pyrosequencing (mean ± SD)	34/50	41–90/20–78	3.97 ± 8.43	3.92 ± 4.54	0.697	Zmetakova I et al. reported no significant difference in methylation of *TIMP3* between patients and healthy controls. Radpour R et al. observed higher methylation level of *TIMP3* in BC patients.
	Radpour R, 2011 [47]	Plasma	EpiTyper assay	36/30	67/na	0.60^b^	0.50^b^	<0.0001	

*MSP* methylation-specific PCR, *QMSP* quantitative methylation-specific PCR, *MS-HRM* methylation-sensitive high-resolution melting, *na* not available

^a^Age indicates mean age or range

^b^Data was extracted from scatter plots or boxplots in the article

^c^ *p* values were calculated by Fisher’s exact test

^d^Nested case–control study; the others are case–control study

Most of these studies investigated tumor suppressor genes and frequently reported the hypermethylation of these genes in BC patients (Table 4 and Additional file 1: Table S2). With ten studies, *RASSF1A* was the most frequently evaluated gene and eight of them reported higher frequency of methylated *RASSF1A* in BC patients compared to controls [42, 44–46, 50, 52, 55–58]. *APC* has been investigated in seven studies [42, 44, 47, 50, 52, 56, 59]. Among them, five studies reported higher frequency of methylated *APC* in plasma/serum DNA of BC patients. Higher frequency of methylated *RARB* (also known as *RARβ2*) was observed in four studies [44, 56, 57, 59]. Methylation levels of *ESR1* [42, 51, 52, 54], *GSTP1* [44, 47, 56], and *TIMP3* [42, 47] were each investigated in two or more studies, but each gene yielded with inconclusive results. Hypermethylation of *SFN* (also known as stratifin or *14-3-3-σ*) [51, 54], *BRCA1* [47, 60], *CST6* [47, 49], and *DAPK* [49, 50] were confirmed in two independent studies, respectively (Table 4). Brooks J.D. et al. [56] reported no significant differences in the methylation of all four genes (*RASSF1A*, *GSTP1*, *APC*, and *RARB*) investigated between BC cases and controls. It is worth to point out that the DNA amounts used in this study were about five times less than the amount hypothetically required to achieve optimal sensitivity and non-specific amplification might occur due to a high number of PCR cycles (i.e., quantitative MSP (QMSP) was run for 50 cycles), as the authors discussed in the article. The authors observed lower frequency of methylation than expected among cases and higher than expected among controls in this study as compared to other studies (review in [66]), which might be the reasons for the negative results. Other genes, which were investigated in only one study, were summarized in Additional file 1: Table S2.

## Discussion

Our literature review identified 45 articles investigating blood-based DNA methylation markers for BC detection or risk evaluation, with DNA isolated from whole blood or from serum or plasma. In this systematic review, we summarized the differences in epigenome-wide DNA methylation levels or gene-specific methylation that were between BC patients and healthy females in all these studies. In particular, several large nested or respective case–control studies were conducted in recent years. This could be partly attributed to the novel emerging techniques, such as Infinium Humanmethylation 27K or 450K array or whole genome bisulfite sequencing (WGBS), which are effective ways to screen for and identify large numbers of methylation markers.

Even though whole blood DNA presents a mixture of leucocytes subtypes, DNA methylation from whole blood samples seems to be promising reservoir for informative biomarkers for BC risk stratification. Two nested case–control studies have concluded that such genomic hypomethylation continuum can be evident at blood DNA level and may identify high-risk women before developing BC [18, 19]. Some retrospective case–control studies also reported that cancer patients have lower global methylation levels in blood DNA compared to controls (Table 2). As blood DNA can be assessed easily, its epigenetic effects on cancer propensity could be repeatedly examined in specified time intervals.

Repetitive DNA sequences (e.g., LINE-1, Alu, and Sat2) are all comparatively rich in CpG dinucleotides and contain a large portion of total methylcytosine levels in the genome [67, 68]. In this regard, some researchers suggested that repetitive elements in blood DNA might be surrogate for genomic hypomethylation. Studies of BC, however, have yielded heterogeneous results (Table 2). Choi et al. [26] found decreased methylation of 5-mdC in blood DNA of women with BC compared to controls; meanwhile, Wu HC et al. [31] and Cho et al. [27] found decreased methylation of Sat2 in BC patients. Xu et al. [29], however, found increased global methylation among cases using the luminometric methylation assay. In the study of Choi et al., LINE-1 methylation and %5-mdC were not correlated, and only hypomethylation quantified as %5-mdC level was significantly associated with BC risk [26]. The inconsistencies between results in BC patients and normal females probably arise from different detection targets, using different techniques and/or from differential distributions of clinical characteristics.

In the implementation and interpretation of studies based on blood samples, a potential limitation deserving particular attention is that differences in methylation profiles might also reflect differences in the proportions of the leukocyte subpopulations that make up the whole blood [69, 70]. Hence, the majority of EWASs adjusted their analysis for leucocyte distribution with the algorithm of Houseman et al. [69]. Nevertheless, even if BC-related methylation patterns were partly due to confounding by leucocyte distribution, they might still be useful as biomarkers of BC.

Circulating cfDNA is defined as extracellular DNA occurring in blood. Both plasma and serum are cell-free blood specimens that were used for the determination of cfDNA. Silencing of tumor suppressor genes by promoter hypermethylation is known to be a frequent and early event in carcinogenesis [11]. Further, changes in methylation patterns observed in tumors are also detectable in cfDNA of women with BC and showed good concordance [50, 71–74]. This makes the possibility of using these alterations candidate markers for early tumor detection. Among all the identified studies in our review, the largest number of studies was found for *BRCA1* and *RASSF1A*, for which higher frequencies of methylated *BRCA1* and *RASSF1A* in BC patients than in healthy females were reported rather consistently. Other tumor suppressor genes, such as *APC*, *RARB*, *GSTP1*, *DAPK*, and *SFN* were also found more frequently methylated in BC cases than in controls. Methylation-specific PCR was the most frequently employed method in the studies evaluating the methylation of specific genes in whole blood and plasma/serum.

Circulating cfDNA, presumably shed from the original primary tumor, can be retrieved and tested for genetic and epigenetic alterations. However, so far, little is known about the relationship between detection of epigenetic abnormalities in primary BC tissue and detection of such abnormalities in plasma or serum. In addition, the amount of cfDNA is around 5–20 ng/ml in the circulation of a normal individual [62, 75], which strongly depends on the accurate sample processing [61]. This could be the main obstacle in finding tumor-specific differences in sera/plasma and the main reason of the lack of sensitivity of the epigenetic biomarkers studied [42]. cfDNA may be released to the circulation via passive release as a result of cellular apoptosis and necrosis and/or active secretion from live cells. The cfDNA can comprise long fragments or shorter fragments ranging from around 20 to 20 kb, depending on their mechanism of release into the circulation [63, 64]. It has been shown by experiments on fetal DNA in the maternal circulation that the half-life of free DNA in blood is only around 16 min [76]. The limited amount, intrinsic characteristics, and short half-life of cell-free DNA could partly explain that for the markers evaluated in more than one study, the methylation differences between cases and controls are not consistent and sometimes varied greatly across studies. The discrepancy probably could also arise from diverse study design, use of different sources of DNA, and/or from differential distributions of clinical characteristics.

Changes in DNA methylation profiles, both at overall genomic level and specific loci, have been associated with BC risk (Fig. 2 and Fig. 3). Among all the studies included in the present review, in total, eight studies measured overall WBC global DNA methylation and BC risk (Fig. 2). Four of these studies [18, 19, 26, 38] have found a significant elevated risk for BC between those in the lowest quantile of global DNA methylation compared to those in the highest methylation quantile. However, Kuchiba A et al. [36] and Xu X et al. [29] found a positive association between LUMA methylation and increased BC risk. The few studies investigating the gene-specific methylation in blood DNA also supported the potential for gene-specific methylation as biomarkers for risk (Fig. 3). However, research in this field is still at an early stage. So far, the number of studies that conducted epigenome-wide studies to detect BC associated genes is very limited. More evidence, including both genome-wide hypomethylation level and gene-specific hypo- and hypermethylation and BC risk, is still needed to collect.

BC is a highly heterogeneous disease. Many of the established risk factors are linked to the development of the disease. The highest risk factor for sporadic BC is increasing age. The incidence of BC in women doubles for every 10 years until menopause with a relative risk of >10-fold [77]. Because promoter hypermethylation may be related to age, studies investigating a potential diagnostic utility for methylated genes should have a reasonable number of age-matched controls. While some authors chose an age-matched control group, others did not and the age difference between cases and controls was often rather large. For example, Zmetakova I et al. [42] compared BC patients with age range between 41 and 90 years with healthy blood donors of considerably younger age (range 20–78). The control group of the study conducted by Ito Y et al. [25] even had an average of 43.2, which is almost 10 years younger than the mean age of BC patients (52.6) they included. Thus, the observed methylation differences and associations between methylation changes and BC risk might be confounded by age.

To our knowledge, this is the first review to systematically and comprehensively review and summarize results of epidemiological studies on the association of DNA methylation in blood with BC. In the interpretation of this review, some limitations have to be considered. Although two widely used databases were searched and cross-referencing of identified articles was applied, we cannot exclude having missed relevant studies. Furthermore, studies were reported in a rather heterogeneous manner, which limited possibilities of a standardized summary of the results. Because of the heterogeneous nature of the included studies and the fact that quite a few markers were evaluated in single studies only, we did not conduct meta-analyses and our tables only provide a narrative summary of the reported methylation differences.

## Conclusions

Our review suggests the possibility of using blood-based methylation markers for risk stratification or the early detection of BC, as a number of studies support an association between methylation changes in blood and BC risk, irrespective of full understanding of the pathophysiological mechanisms. However, the evidence is still very limited. Optimized marker panels are yet to be developed and promising candidate markers needed to be validated in prospective study cohorts and tested in large screening populations by high quality studies. In addition, there is a strong need for large, methodologically rigorous epidemiological studies to figure out the potential role of methylation changes in blood in breast carcinogenesis and their implications for detection. Especially, the investigation of methylation changes in cfDNA holds great promises. Here, optimization of methods for genome-wide methylation analysis of small amounts of DNA is needed.

## Additional file

Additional file 1: Table S1. Gene-specific methylation in peripheral blood in breast cancer cases and controls investigated in only one study. **Table S2**. Specific-gene methylation in serum or plasma DNA in breast cancer cases and controls investigated in only one study. Supplementary materials. Exclusion reasons in full-text selection procedure. (DOCX 96 kb)

## Abbreviations

5-mdC: 5-Methyldeoxycytosine
BC: Breast cancer
CA15-3: Cancer antigen 15-3
CA27-29: Cancer antigen 27-29
cfDNA: Cell-free DNA
LC-MS: Liquid chromatography-mass spectrometry
LINE-1: Long interspersed nuclear element-1
LUMA: Luminometric methylation assay
MSP: Methylation-specific PCR
OR: Odds ratio
QMSP: Quantitative methylation-specific PCR
Sat2: Satellite 2
