# Supplementary material for: Blood-based DNA methylation as biomarker for breast cancer: a systematic review
Source: Clin Epigenetics. 2016 Nov 14;8:115. doi: 10.1186/s13148-016-0282-6 (PMC5109688; doi:10.1186/s13148-016-0282-6)
Supplement: Supplementary file 1 — Gene-specific methylation in peripheral blood in breast cancer cases and controls investigated in only one study. Table S2. Specific-gene methylation in serum or plasma DNA in breast cancer cases and controls investigated in only one study. Supplementary materials. Exclusion reasons in full-text selection procedure. (DOCX 96 kb) [file 13148_2016_282_MOESM1_ESM.docx]

**Additional files**

| **Additional file 1:Table S1** Gene-specific methylation in peripheral blood in breast cancer cases and controls investigated in only one study | | | | | | | |
| --- | --- | --- | --- | --- | --- | --- | --- |
| Gene | Author, year [ref] | Assay (value) | Cases No./Control No. | Case age/ Control age (y)* | Meth (case) | Meth (control) | *p* value |
| *PLAGL1* | [[1](#_ENREF_1)] | pyrosequencing (mean ± SD ) | 189/363 | 56/56 | 55.92 ± 5.08 | 55.91 ±4.92 | 0.932 |
| *H19* | [[1](#_ENREF_1)] | pyrosequencing (mean ± SD ) | 189/363 | 56/56 | 60.10 ±3.97 | 59.59 ±4.35 | 0.171 |
| *KvDMR* | [[1](#_ENREF_1)] | pyrosequencing (mean ± SD ) | 189/363 | 56/56 | 44.13 ± 2.36 | 44.17 ±2.34 | 0.860 |
| *RB1* | [[1](#_ENREF_1)] | pyrosequencing (mean ± SD ) | 189/363 | 56/56 | 68.68± 5.38 | 68.61 ±4.30 | 0.772 |
| *SNRPN* | [[1](#_ENREF_1)] | pyrosequencing (mean ± SD ) | 189/363 | 56/56 | 43.93 ±2.91 | 44.03 ± 3.03 | 0.932 |
| *PEG3* | [[1](#_ENREF_1)] | pyrosequencing (mean ± SD ) | 189/363 | 56/56 | 52.57 ±2.89 | 52.05 ± 2.93 | **0.042** |
| *PTEN* | [[2](#_ENREF_2)] | MSP (%) | 103/50 | 49.8/36.6 | 41.7 | 6 | **< 0.001** |
| *KILLIN/PTEN* | [[3](#_ENREF_3)] | MSP (%) | 23/20 | 51.4/49.7 | 28 | 24 | 0.111 |
| *HYAL2* | [[4](#_ENREF_4)] | Infinium 27K Array (Discovery) (mean and IQR) | 72/24 | 47/48.5 | 0.65 (0.59-0.69) | 0.50 (0.47-0.57) | **< 0.0001** |
|  |  | MassARRAY (Validation I ) (mean and IQR) | 338/507 | 46.1/44.7 | 0.66 (0.59-0.73) | 0.54 (0.48-0.60) | **< 0.0001** |
|  |  | MassARRAY (Validation II ) (mean and IQR) | 189/189 | 59.6/61.2 | 0.64 (0.58-0.70) | 0.50 (0.46-0.54) | **< 0.0001** |
| *ADAM23* | [[5](#_ENREF_5)] | pyrosequencing (mean ± SD ) | 34/50 | 41-90/20-78 | 2.29 ± 0.58 | 2.18 ± 0.39 | 0.511 |
| *CXCL12* | [[5](#_ENREF_5)] | pyrosequencing (mean ± SD ) | 34/50 | 41-90/20-78 | 2.79 ± 0.84 | 2.28 ± 0.50 | **0.044** |
| *PGRB* | [[5](#_ENREF_5)] | pyrosequencing (mean ± SD ) | 34/50 | 41-90/20-78 | 5.41 ± 2.03 | 4.32 ± 1.13 | 0.056 |
| *BRMS1* | [[5](#_ENREF_5)] | pyrosequencing (mean ± SD ) | 34/50 | 41-90/20-78 | 1.56 ± 0.93 | 1.40 ± 0.70 | 0.726 |
| *SOCS1* | [[5](#_ENREF_5)] | pyrosequencing (mean ± SD ) | 34/50 | 41-90/20-78 | 1.09 ± 0.38 | 0.96 ± 0.28 | 0.271 |
| *p21/CIP1* | [[6](#_ENREF_6)] | MSP (%) | 125/100 | na/na | 34.66 | 2.6 | 0.000 |
| *DBC2* | [[7](#_ENREF_7)] | nested MSP (%) | 50/30 | 55.7/38 | 46 | 16.6 | **0.007** |
| *HIN1* | [[8](#_ENREF_8)] | MethyLight (%) | 40/40 | 50.8/48.3 | 10.00 | 5.00 | > 0.05 |
| *TWIST1* | [[8](#_ENREF_8)] | MethyLight (%) | 40/40 | 50.8/48.3 | 0.00 | 0.00 | > 0.05 |
| *CYCLIND2* | [[8](#_ENREF_8)] | MethyLight (%) | 40/40 | 50.8/48.3 | 0.00 | 3.00 | > 0.05 |

| **Table S1** continued | | | | | | | |
| --- | --- | --- | --- | --- | --- | --- | --- |
| Gene | Author, year [ref] | Assay (value) | Cases No./Control No. | Case age/ Control age (y)* | Meth (case) | Meth (control) | *p* value |
| *BRIP1* | [[9](#_ENREF_9)] | MethyLight (%) | 306/653 | 50-74/50-74 | 31 | 30.5 | 0.852 |
| *SIRT3* | [[9](#_ENREF_9)] | MethyLight (%) | 294/627 | 50-74/50-74 | 20.7 | 16.3 | 0.1 |
| *NUP155* | [[9](#_ENREF_9)] | MethyLight (%) | 306/653 | 50-74/50-74 | 21.9 | 28.6 | **0.031** |
| *PITX2(I)* | [[9](#_ENREF_9)] | MethyLight (%) | 320/676 | 50-74/50-74 | 33.1 | 38 | 0.133 |
| *PITX2 (II)* | [[9](#_ENREF_9)] | MethyLight (%) | 320/676 | 50-74/50-74 | 48.1 | 48.5 | 0.916 |
| *DCC* | [[9](#_ENREF_9)] | MethyLight (%) | 302/638 | 50-74/50-74 | 35.1 | 41.7 | 0.059 |
| *ZNF217* | [[9](#_ENREF_9)] | MethyLight (%) | 302/638 | 50-74/50-74 | 39.4 | 48.9 | **0.005** |
| *FLJ39739* | [[9](#_ENREF_9)] | MethyLight (%) | 294/627 | 50-74/50-74 | 52 | 49.4 | 0.474 |
| *PGR* | [[9](#_ENREF_9)] | MethyLight (%) | 320/676 | 50-74/50-74 | 69.7 | 70.7 | 0.754 |
| *CDH13* | [[9](#_ENREF_9)] | MethyLight (%) | 320/676 | 50-74/50-74 | 13.8 | 15.4 | 0.485 |
| *HSD17B4* | [[9](#_ENREF_9)] | MethyLight (%) | 320/676 | 50-74/50-74 | 17.5 | 14.3 | 0.19 |
| *PTGS2* | [[9](#_ENREF_9)] | MethyLight (%) | 320/676 | 50-74/50-74 | 71.3 | 77.1 | **0.049** |
| *SLC6A20* | [[9](#_ENREF_9)] | MethyLight (%) | 320/676 | 50-74/50-74 | 0.9 | 1.8 | 0.307 |
| *NEUROG1* | [[9](#_ENREF_9)] | MethyLight (%) | 302/638 | 50-74/50-74 | 4.3 | 4.7 | 0.783 |
| *HOXA1* | [[9](#_ENREF_9)] | MethyLight (%) | 306/653 | 50-74/50-74 | 11.1 | 13.9 | 0.247 |
| *TITF1* | [[9](#_ENREF_9)] | MethyLight (%) | 320/676 | 50-74/50-74 | 13.8 | 19.5 | **0.025** |
| *GDNF* | [[9](#_ENREF_9)] | MethyLight (%) | 306/653 | 50-74/50-74 | 14.7 | 18.7 | 0.147 |
| *NEUROD1* | [[9](#_ENREF_9)] | MethyLight (%) | 298/642 | 50-74/50-74 | 30.5 | 38.9 | **0.014** |
| *SFRP1* | [[9](#_ENREF_9)] | MethyLight (%) | 320/676 | 50-74/50-74 | 29.4 | 37.4 | **0.014** |
| *MYOD1* | [[9](#_ENREF_9)] | MethyLight (%) | 320/676 | 50-74/50-74 | 60 | 63.5 | 0.244 |
| *CYP1B1* | [[9](#_ENREF_9)] | MethyLight (%) | 320/676 | 50-74/50-74 | 7.5 | 4.9 | 0.096 |
| *SEZ6L* | [[9](#_ENREF_9)] | MethyLight (%) | 306/653 | 50-74/50-74 | 50.3 | 52.8 | 0.482 |

Abbreviations: MSP: methylation specific PCR; q-MSP, quantitative methylatin specific PCR; MS-HRM: methylation-sensitive high-resolution melting; na, not available.

| **Additional file 2: Table S2** Specific-gene methylation in serum or plasma DNA in breast cancer cases and controls investigated in only one study | | | | | | | | |
| --- | --- | --- | --- | --- | --- | --- | --- | --- |
| Gene | Author, year [ref] | Sample | Assay (value) | Cases No./Control No. | Case age/ Control age (y)* | Meth (case) | Meth (control) | *p* value |
| FHIT | [[10](#_ENREF_10)] | serum | Bisulfite sequencing PCR and MS-HRM (%) | 36^a^/30^a^ | na/na | 64.2 | 35.3 | **<0.05** |
| KILLIN/PTEN | [[3](#_ENREF_3)] | plasma | MSP | 4/20 | 51.4/49.7 | 6 | 40 | **<0.05** |
| *ITIH5* | [[11](#_ENREF_11)] | serum | MS-PCR (%) | 250/237 | 33-86/33-86 | 18.9 | 5.2 | - |
| *DKK3* | [[11](#_ENREF_11)] | serum | MS-PCR (%) | 250/237 | 33-86/33-86 | 31.35 | 1.25 | - |
| *WIF1* | [[11](#_ENREF_11)] | serum | MS-PCR (%) | 112/102 | 36-87/36-87 | 35 | 5 | - |
| *SFRP1* | [[11](#_ENREF_11)] | serum | MS-PCR (%) | 112/102 | 36-87/36-87 | 11 | 10 | - |
| *SFRP2* | [[11](#_ENREF_11)] | serum | MS-PCR (%) | 112/102 | 36-87/36-87 | 19 | 12 | - |
| *ADAM23* | [[5](#_ENREF_5)] | plasma | pyrosequencing (mean ± SD) | 34/50 | 41-90/20-78 | 3.82 ± 7.33 | 2.69 ± 1.50 | 0.073 |
| *CXCL12* | [[5](#_ENREF_5)] | plasma | pyrosequencing (mean ± SD) | 34/50 | 41-90/20-78 | 2.85 ± 2.84 | 2.68 ± 1.92 | 0.879 |
| *PGRB* | [[5](#_ENREF_5)] | plasma | pyrosequencing (mean ± SD) | 34/50 | 41-90/20-78 | 2.97 ± 2.15 | 4.69 ± 5.85 | 0.206 |
| *CDH1* | [[5](#_ENREF_5)] | plasma | pyrosequencing (mean ± SD) | 34/50 | 41-90/20-78 | 6.53 ± 5.32 | 7.73 ± 3.60 | **0.045** |
| *SYK* | [[5](#_ENREF_5)] | plasma | pyrosequencing (mean ± SD) | 34/50 | 41-90/20-78 | 3.00 ± 5.65 | 2.14 ± 2.28 | 0.488 |
| *BRMS1* | [[5](#_ENREF_5)] | plasma | pyrosequencing (mean ± SD) | 34/50 | 41-90/20-78 | 3.12 ± 2.36 | 2.56 ± 1.57 | 0.516 |
| *SOCS1* | [[5](#_ENREF_5)] | plasma | pyrosequencing (mean ± SD) | 34/50 | 41-90/20-78 | 1.76 ± 0.90 | 2.08 ± 1.91 | 0.358 |
| *SLC19A3* | [[12](#_ENREF_12)] | plasma | MSRED-qPCR (relative level) | 60/60 | 58/57 | 0.62^b^ | 0.35^b^ | **<0.0001** |
| *BIN1* | [[13](#_ENREF_13)] | plasma | EpiTyper assay (mean) | 36/30 | 67/na | 0.69^b^ | 0.51^b^ | **<0.0001** |
| *BMP6* | [[13](#_ENREF_13)] | plasma | EpiTyper assay (mean) | 36/30 | 67/na | 0.65^b^ | 0.55^b^ | 0.068 |
| *ESR-b* | [[13](#_ENREF_13)] | plasma | EpiTyper assay (mean) | 36/30 | 67/na | 0.64^b^ | 0.56^b^ | 0.122 |
| *P16* | [[13](#_ENREF_13)] | plasma | EpiTyper assay (mean) | 36/30 | 67/na | 0.95^b^ | 0.78^b^ | **0.001** |
| *P21* | [[13](#_ENREF_13)] | plasma | EpiTyper assay (mean) | 36/30 | 67/na | 0.65^b^ | 0.41^b^ | **<0.0001** |
| *TWIST* | [[14](#_ENREF_14)] | serum | QM-MSP (%) | 119/125 | 51/51 | 54.6 | 8.0 | **<0.001** |
| *HIN-1* | [[14](#_ENREF_14)] | serum | QM-MSP (%) | 119/125 | 51/51 | 30.3 | 0 | **0.006** |
| *Hsulf-1* | [[15](#_ENREF_15)] | serum | MSP (%) | 21/21 | 58-70/na | 76.2 | 19.0 | **<0.05** |
| *ATM* | [[16](#_ENREF_16)] | plasma | Methylight (%) | 50/9 | na/na | 14 | 0 | **<0.05** |

Abbreviations: MSP: methylation specific PCR; q-MSP, quantitative methylatin specific PCR; MS-HRM: methylation-sensitive high-resolution melting; na, not available.

^a^ Only data from breast ductal cancer and healthy controls was extracted and included in the table.

^b^ Data was extracted from scatter plots or boxplots in the article.

**Reference**

1. Harrison K, Hoad G, Scott P, Simpson L, Horgan GW, Smyth E et al. Breast cancer risk and imprinting methylation in blood. Clin Epigenetics 2015; 7(1):92. doi:10.1186/s13148-015-0125-x.

2. Yari K, Payandeh M, Rahimi Z. Association of the hypermethylation status of PTEN tumor suppressor gene with the risk of breast cancer among Kurdish population from Western Iran. Tumour Biol 2015. doi:10.1007/s13277-015-4731-1.

3. Ng EK, Shin VY, Leung CP, Chan VW, Law FB, Siu MT et al. Elevation of methylated DNA in KILLIN/PTEN in the plasma of patients with thyroid and/or breast cancer. Onco Targets Ther 2014; 7:2085-2092. doi:10.2147/ott.s53597.

4. Yang R, Pfutze K, Zucknick M, Sutter C, Wappenschmidt B, Marme F et al. DNA methylation array analyses identified breast cancer-associated HYAL2 methylation in peripheral blood. Int J Cancer 2015; 136(8):1845-1855. doi:10.1002/ijc.29205.

5. Zmetakova I, Danihel L, Smolkova B, Mego M, Kajabova V, Krivulcik T et al. Evaluation of protein expression and DNA methylation profiles detected by pyrosequencing in invasive breast cancer. Neoplasma 2013; 60(6):635-646. doi:10.4149/neo_2013_082.

6. Askari M, Sobti RC, Nikbakht M, Sharma SC. Aberrant promoter hypermethylation of p21 (WAF1/CIP1) gene and its impact on expression and role of polymorphism in the risk of breast cancer. Molecular and Cellular Biochemistry 2013; 382(1-2):19-26. doi:10.1007/s11010-013-1696-5.

7. Hajikhan Mirzaei M, Noruzinia M, Karbassian H, Shafeghati Y, Keyhanee M, Bidmeshki-Pour A. Evaluation of Methylation Status in the 5'UTR Promoter Region of the DBC2 Gene as a Biomarker in Sporadic Breast Cancer. Cell J 2012; 14(1):19-24.

8. Cho YH, Yazici H, Wu HC, Terry MB, Gonzalez K, Qu M et al. Aberrant promoter hypermethylation and genomic hypomethylation in tumor, adjacent normal tissues and blood from breast cancer patients. Anticancer Res 2010; 30(7):2489-2496.

9. Widschwendter M, Apostolidou S, Raum E, Rothenbacher D, Fiegl H, Menon U et al. Epigenotyping in peripheral blood cell DNA and breast cancer risk: a proof of principle study. PLoS One 2008; 3(7):e2656. doi:10.1371/journal.pone.0002656.

10. Liu L, Sun L, Li C, Li X, Zhang Y, Yu Y et al. Quantitative detection of methylation of FHIT and BRCA1 promoters in the serum of ductal breast cancer patients. Biomed Mater Eng 2015; 26 Suppl 1:S2217-2222. doi:10.3233/bme-151527.

11. Kloten V, Becker B, Winner K, Schrauder MG, Fasching PA, Anzeneder T et al. Promoter hypermethylation of the tumor-suppressor genes ITIH5, DKK3, and RASSF1A as novel biomarkers for blood-based breast cancer screening. Breast Cancer Res 2013; 15(1):R4. doi:10.1186/bcr3375.

12. Ng EK, Leung CP, Shin VY, Wong CL, Ma ES, Jin HC et al. Quantitative analysis and diagnostic significance of methylated SLC19A3 DNA in the plasma of breast and gastric cancer patients. PLoS One 2011; 6(7):e22233. doi:10.1371/journal.pone.0022233.

13. Radpour R, Barekati Z, Kohler C, Lv Q, Burki N, Diesch C et al. Hypermethylation of tumor suppressor genes involved in critical regulatory pathways for developing a blood-based test in breast cancer. PLoS One 2011; 6(1):e16080. doi:10.1371/journal.pone.0016080.

14. Kim JH, Shin MH, Kweon SS, Park MH, Yoon JH, Lee JS et al. Evaluation of promoter hypermethylation detection in serum as a diagnostic tool for breast carcinoma in Korean women. Gynecol Oncol 2010; 118(2):176-181. doi:10.1016/j.ygyno.2010.04.016.

15. Chen Z, Fan JQ, Li J, Li QS, Yan Z, Jia XK et al. Promoter hypermethylation correlates with the Hsulf-1 silencing in human breast and gastric cancer. Int J Cancer 2009; 124(3):739-744. doi:10.1002/ijc.23960.

16. Papadopoulou E, Davilas E, Sotiriou V, Georgakopoulos E, Georgakopoulou S, Koliopanos A et al. Cell-free DNA and RNA in plasma as a new molecular marker for prostate and breast cancer. Ann N Y Acad Sci 2006; 1075:235-243. doi:10.1196/annals.1368.032.

**Additional file 3: Supplementary materials.** Exclusion reasons in full-text selection procedure

**Studies excluded from this review because of:**

***(A)Studies with no eligible controls (n=21)***

(1) Hansmann, T., et al., *Constitutive promoter methylation of BRCA1 and RAD51C in patients with familial ovarian cancer and early-onset sporadic breast cancer.* Hum Mol Genet, 2012. **21**(21): p. 4669-79.

(2) Fridrichova, I., et al., *CXCL12 and ADAM23 hypermethylation are associated with advanced breast cancers.* Transl Res, 2015. **165**(6): p. 717-30.

(3) Bae, Y.K., et al., *Gene promoter hypermethylation in tumors and plasma of breast cancer patients.* Cancer Res Treat, 2005. **37**(4): p. 233-40.

(4) Pang, D., et al., *Methylation profiles of the BRCA1 promoter in hereditary and sporadic breast cancer among Han Chinese.* Med Oncol, 2012. **29**(3): p. 1561-8.

(5) Wojdacz, T.K., et al., *Methylation of cancer related genes in tumor and peripheral blood DNA from the same breast cancer patient as two independent events.* Diagn Pathol, 2011. **6**: p. 116.

(6) Chimonidou, M., et al., *Breast cancer metastasis suppressor-1 promoter methylation in primary breast tumors and corresponding circulating tumor cells.* Mol Cancer Res, 2013. **11**(10): p. 1248-57.

(7) Gawish, H.H., H.A. Hagrass, and E.H.A. Bary, *RASSF1A Gene Hypermethylation in Tissue and Serum Together with Tissue Protein Expression in Breast Cancer Patients.* Life Science Journal-Acta Zhengzhou University Overseas Edition, 2012. **9**(3): p. 667-675.

(8) Kim, G.E., et al., *Detection of Slit2 promoter hypermethylation in tissue and serum samples from breast cancer patients.* Virchows Arch, 2011. **459**(4): p. 383-90.

(9) Sturgeon, S.R., et al., *Detection of promoter methylation of tumor suppressor genes in serum DNA of breast cancer cases and benign breast disease controls.* Epigenetics, 2012. **7**(11): p. 1258-67.

(10) Sharma, G., et al., *CpG hypomethylation of MDR1 gene in tumor and serum of invasive ductal breast carcinoma patients.* Clin Biochem, 2010. **43**(4-5): p. 373-9.

(11) Mirza, S., et al., *Promoter hypermethylation of TMS1, BRCA1, ERalpha and PRB in serum and tumor DNA of invasive ductal breast carcinoma patients.* Life Sci, 2007. **81**(4): p. 280-7.

(12) Sharma, G., et al., *Clinical significance of promoter hypermethylation of DNA repair genes in tumor and serum DNA in invasive ductal breast carcinoma patients.* Life Sci, 2010. **87**(3-4): p. 83-91.

(13) Shukla, S., et al., *Detection of RASSF1A and RARbeta hypermethylation in serum DNA from breast cancer patients.* Epigenetics, 2006. **1**(2): p. 88-93.

(14) Hagrass, H.A., et al., *Methylation status and protein expression of RASSF1A in breast cancer patients.* Mol Biol Rep, 2014. **41**(1): p. 57-65.

(15) Hu, X.C., I.H. Wong, and L.W. Chow, *Tumor-derived aberrant methylation in plasma of invasive ductal breast cancer patients: clinical implications.* Oncol Rep, 2003. **10**(6): p. 1811-5.

(16) Zhang, Y., et al., *Hypomethylation of DNA-binding inhibitor 4 serves as a potential biomarker in distinguishing acquired tamoxifen-refractory breast cancer.* Int J Clin Exp Pathol, 2015. **8**(8): p. 9500-5.

(17) Sharma, G., et al., *Promoter hypermethylation of p16INK4A, p14ARF, CyclinD2 and Slit2 in serum and tumor DNA from breast cancer patients.* Life Sci, 2007. **80**(20): p. 1873-81.

(18) Hagrass, H.A., H.F. Pasha, and A.M. Ali, *Estrogen receptor alpha (ER alpha) promoter methylation status in tumor and serum DNA in Egyptian breast cancer patients.* Gene, 2014. **552**(1): p. 81-86.

(19) Jing, F., et al., *CpG island methylator phenotype of multigene in serum of sporadic breast carcinoma.* Tumour Biol, 2010. **31**(4): p. 321-31.

(20) Sebova, K., et al., *RASSF1A and CDH1 hypermethylation as potential epimarkers in breast cancer.* Cancer Biomark, 2011. **10**(1): p. 13-26.

(21) Chimonidou, M., et al., *SOX17 promoter methylation in circulating tumor cells and matched cell-free DNA isolated from plasma of patients with breast cancer.* Clin Chem, 2013. **59**(1): p. 270-9.

***(B)Studies with no eligible breast cancer cases, with only patients with benign breast diseases (n=1)***

(1) Rykova, E.Y., et al., *Methylation-based analysis of circulating DNA for breast tumor screening.* Ann N Y Acad Sci, 2008. **1137**: p. 232-5.

***(C)Studies investigated the methylation of specific genes in CTCs isolated from blood (n=1)***

(1) Chimonidou, M., et al., *DNA methylation of tumor suppressor and metastasis suppressor genes in circulating tumor cells.* Clin Chem, 2011. **57**(8): p. 1169-77.

***(D)Methylation levels and ORs could not be extracted (n=5)***

(1) Anjum, S., et al., *A BRCA1-mutation associated DNA methylation signature in blood cells predicts sporadic breast cancer incidence and survival.* Genome Med, 2014. **6**(6): p. 47.

(2) Xu, Z., et al., *Epigenome-wide association study of breast cancer using prospectively collected sister study samples.* J Natl Cancer Inst, 2013. **105**(10): p. 694-700.

(3) Rykova, E.Y., et al., *Breast cancer diagnostics based on extracellular DNA and RNA circulating in blood.* Biomeditsinskaya Khimiya, 2008. **54**(1): p. 94-103.

(4) Heyn, H., et al., *DNA methylation profiling in breast cancer discordant identical twins identifies DOK7 as novel epigenetic biomarker.* Carcinogenesis, 2013. **34**(1): p. 102-8.

(5) Fu, A., et al., *Genetic and epigenetic associations of circadian gene TIMELESS and breast cancer risk.* Mol Carcinog, 2012. **51**(12): p. 923-9.

***(E)Cases are from a twin pair study cohort with different cancer types (n=1)***

(1) Roos, L., et al., *Integrative DNA methylome analysis of pan-cancer biomarkers in cancer discordant monozygotic twin-pairs.* Clin Epigenetics, 2016. **8**: p. 7.
